# Effects of previous steroid treatment on ischemic stroke outcomes: a propensity score-matched hospital analysis

**DOI:** 10.3389/fphar.2025.1615170

**Published:** 2025-06-17

**Authors:** Yasser Alatawi, Eman A. Alraddadi, Abdullah A. Alhifany, Faisal E. Alzahrani, Saud M. Alknawy, Nawaf M. Aljohani, Husun K. Kecheck, Alanoud K. Alaslab, Aser F. Alamri, Ahmed Aljabri, Daniyah A. Almarghalani, Faisal F. Alamri

**Affiliations:** ^1^ Department of Pharmacy Practice, Faculty of Pharmacy, University of Tabuk, Tabuk, Saudi Arabia; ^2^ Department of Basic Sciences, College of Science and Health Professions, King Saud Bin Abdulaziz University for Health Sciences, Jeddah, Saudi Arabia; ^3^ King Abdullah International Medical Research Center, Jeddah, Saudi Arabia; ^4^ Pharmaceutical Practices Department, College of Pharmacy, Umm Al-Qura University, Makkah, Saudi Arabia; ^5^ College of Medicine, King Saud Bin Abdulaziz University for Health Sciences, Jeddah, Saudi Arabia; ^6^ College of Medicine, King Saud Bin Abdulaziz University for Health Sciences, Riyadh, Saudi Arabia; ^7^ King Abdullah International Medical Research Center, Riyadh, Saudi Arabia; ^8^ Department of Pharmacy Practice, Faculty of Pharmacy, King Abdulaziz University, Jeddah, Saudi Arabia; ^9^ Department of Pharmacology and Toxicology, College of Pharmacy, Taif University, Taif, Saudi Arabia; ^10^ Stroke Research Unit, Taif University, Taif, Saudi Arabia

**Keywords:** stroke recurrence, steroid exposure, ischemic stroke, propensity score matching, stroke outcome

## Abstract

**Introduction:**

Stroke is a leading cause of morbidity and mortality worldwide, and its recurrence poses significant challenges to patient management and healthcare systems. This hospital-based retrospective observational study investigated the association between prior exposure to systemic corticosteroids and stroke recurrence within the Saudi Arabian population.

**Methods:**

A multicenter retrospective study included acute-subacute adult ischemic stroke patients. Propensity score matching (PSM) was applied to balance baseline characteristics between the steroid and non-steroid groups. The primary outcome was the incidence of stroke recurrence within 365 days of the index stroke. Secondary outcomes included stroke severity and functional independence on admission and discharge, hemorrhagic transformation within 30 days, and mortality rate within 365 days of the index stroke.

**Results:**

Out of 925 patients, 85 (9.19%) received steroids. After PSM, the analysis included 254 patients, with 33.46% in the steroid group and 66.54% in the control group. Steroid-exposed patients had significantly lower National Institutes of Health Stroke Scale (NIHSS) scores at both admission (median 5 [interquartile range (IQR): 1–8] vs 6 [IQR: 3–10], *p* = 0.0087) and discharge (median 1 [IQR: 0–4.5] vs 4 [IQR: 2–9], *p* = 0.0001), but higher modified Rankin Scale (mRS) scores at discharge (median 5 [IQR: 4–5] vs 4 [IQR: 3–5], *p* = 0.0004). Univariate analysis revealed significant associations between steroid exposure and a reduced likelihood of aphasia (OR: 0.33, 95% CI: 0.17–0.67, *p* = 0.0020) and dysarthria (OR: 0.51, 95% CI: 0.30–0.88, *p* = 0.0149). Conversely, steroid exposure was linked to increased risks of pneumonia (OR: 2.08, 95% CI: 1.22–3.55, *p* = 0.0071), deep vein thrombosis-pulmonary embolism (DVT-PE) (OR: 2.70, 95% CI: 1.30–5.62, *p* = 0.0079), and impaired consciousness (OR: 1.80, 95% CI: 1.06–3.04, *p* = 0.0303). In the multivariate analysis, steroid exposure was associated with an increased risk of stroke recurrence (OR: 1.98, 95% CI: 1.01–3.87, *p* = 0.0471). However, this association did not retain significance after adjusting for confounders (OR: 1.14, 95% CI: 0.44–2.96, p = 0.7874).

**Conclusion:**

The study revealed that steroids were associated with significantly lower stroke severity but higher mRS scores. However, the risk of stroke recurrence was similar between the two groups. Moreover, the use of steroids may increase the risk of complications in stroke patients, such as pneumonia and DVT-PE. Future studies with larger sample sizes and more detailed data on steroid use and stroke outcomes are required. These studies would provide more definitive insights and guide clinical decision-making regarding the use of steroids in stroke management.

- Stroke recurrence remains a major global health concern with high disability and mortality rates, necessitating novel therapeutic approaches to improve secondary prevention strategies.
- The impact of systemic steroid exposure prior to ischemic stroke on long-term stroke recurrence and mortality in Saudi patients has not been investigated.
- This study examined the association between prior steroid exposure and the risk of stroke recurrence and mortality rate within 1 year of the index stroke, and complications during admission and hemorrhagic transformation within 1 month were investigated.
- Steroid-exposed stroke patients had lower NIHSS scores at admission and discharge but higher mRS scores, with reduced aphasia and dysarthria risks, yet increased risks of pneumonia, DVT-PE, and impaired consciousness, while the initially observed association between steroids and stroke recurrence lost significance after adjusting for confounders.
- Future research should focus on prospective studies with larger, diverse cohorts to validate findings, explore steroid types, dosing, and alternative therapies, and optimize personalized stroke management strategies.

## 1 Introduction

Stroke recurrence is a significant global health concern, contributing to high rates of disability and mortality. The rate of stroke recurrence ranges from 5.7% to 51.3% and has remained unchanged over the past 2 decades despite advances in medical treatment and secondary prevention strategies (Kolmos et al., 2021). Preventing stroke recurrence is crucial for improving long-term patient outcomes (Feigin et al., 2017). Therefore, identifying novel therapeutic approaches is essential for developing more effective strategies for secondary prevention.

Inflammation plays a pivotal role in the pathophysiology of stroke, influencing both the acute phase and long-term recovery (Iadecola and Anrather, 2011; Alharbi et al., 2022). It is an important contributor to thrombosis, atherosclerosis, and cerebral small vessel disease, key mechanisms that elevate the risk of recurrent stroke (Endres et al., 2022). Consequently, targeting inflammation has emerged as a promising approach for secondary stroke prevention. Anti-inflammatory drugs, such as steroids, have the potential to modulate the inflammatory response associated with stroke (Endres et al., 2022). This underscores the importance of further investigating their role in mitigating stroke recurrence.

Steroids are widely used for their anti-inflammatory effects in various medical conditions, including asthma, chronic obstructive pulmonary disease, rheumatoid arthritis, eczema, inflammatory bowel diseases, lupus, and multiple sclerosis (Möhlmann et al., 2024; Barnes, 2011). However, their impact on stroke outcomes, particularly recurrence, remains poorly understood (Coveney et al., 2020). Moreover, glucocorticoids are associated with side effects, such as hypertension (HTN), hyperglycemia, and lipid abnormalities, which are known risk factors for stroke (Fardet and Fève, 2014). A deeper understanding of the relationship between steroid use and stroke recurrence could refine treatment protocols and contribute to improved patient care.

The unique demographic and epidemiological profile of Saudi Arabia, characterized by high rates of diabetes mellitus (DM), HTN, and dyslipidemia, provides a valuable context for exploring the impact of steroid exposure on stroke recurrence (Al-Nozha et al., 2004; Alraddadi et al., 2024). By focusing on a cohort from Saudi Arabia, the present study aimed to provide insights that are both locally relevant and globally significant. To this end, the primary objective of this study was to examine the association between prior steroid exposure and the risk of stroke recurrence, whereas the secondary objective was to assess baseline characteristics, complications during admission, hemorrhagic transformation within 1 month, and mortality rate within 1 year of the index stroke. To ensure a robust and unbiased comparison, a propensity score matching (PSM) technique was employed to balance the baseline characteristics between patients exposed to steroids and those in the control group, thereby isolating the real impact of steroids on stroke recurrence (Austin, 2011).

## 2 Methods

### 2.1 Study design and setting

This hospital-based retrospective observational study was conducted at two tertiary hospitals: one located in Jeddah (western region) and the other in Riyadh (central region), covering the period from January 2016 to September 2022. The study was approved by the King Abdullah International Medical Research Center (KAIMRC) Institutional Review Board (NRC24R/115/02). As this was an observational study utilizing de-identified retrospective data, the requirement for informed consent was waived.

### 2.2 Participants

**FIGURE 1:**
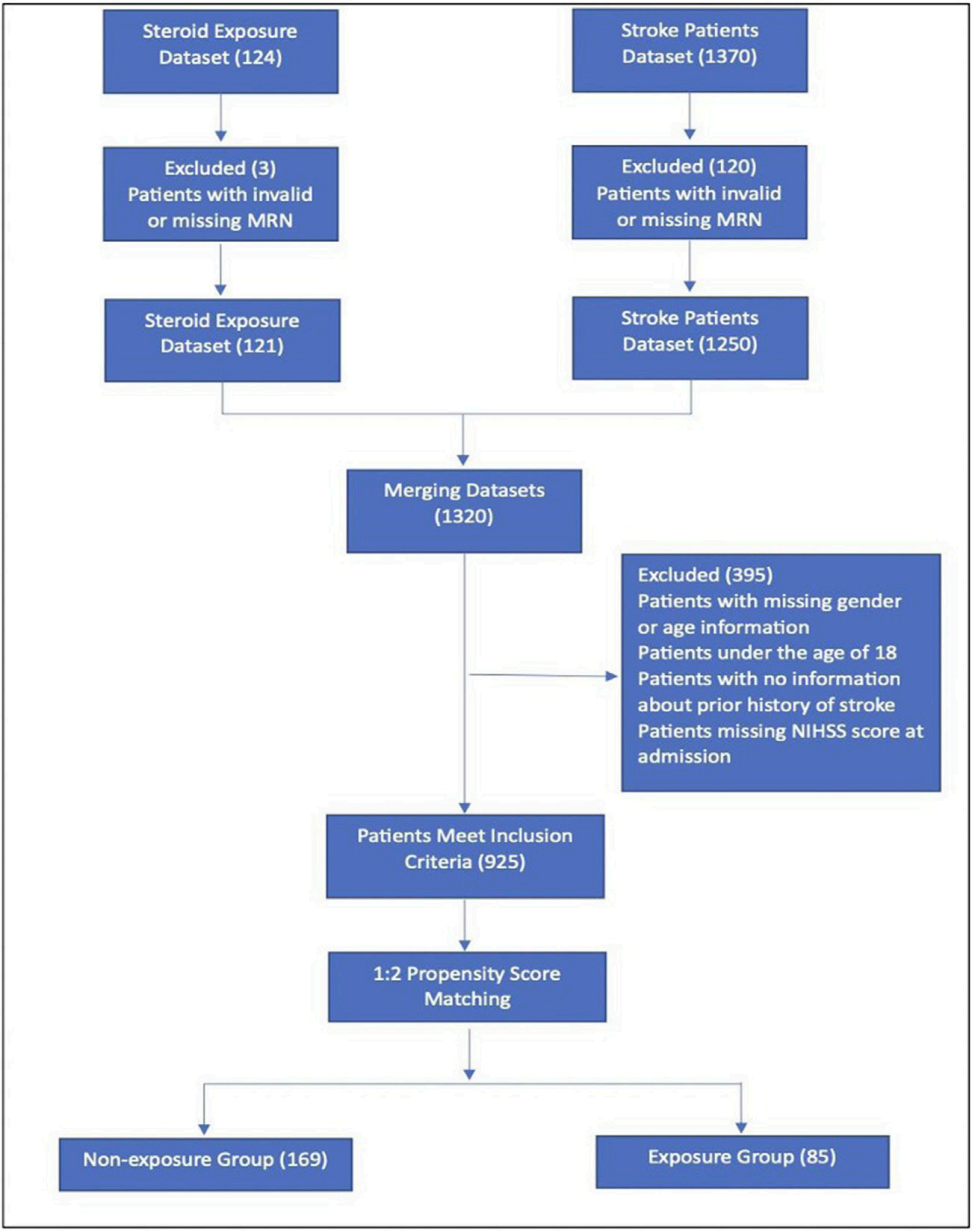
Flowchart of study patients. MRN, medical record number; NIHSS, National Institutes of Health Stroke Scale.

Eligible patients were identified using an electronic medical records system (BESTCare^®^ 2.0) based on predefined inclusion and exclusion criteria, Figure 1. This study included adult patients (aged ≥18 years) who experienced a new ischemic stroke during the study period. Patients were excluded if they had a history of hemorrhagic stroke or transient ischemic attack (TIA), were missing demographic data, such as gender or age, were under 18 years old, or lacked data on prior stroke history or the National Institutes of Health Stroke Scale (NIHSS) scores at admission. The final sample comprised patients with complete data on steroid exposure and relevant covariates.

### 2.3 Exposure and outcome measures

The primary exposure of interest was a previous exposure to steroids, defined as any documented use of systemic corticosteroids within 7 days preceding the index stroke. The primary outcome was stroke recurrence, described as a new ischemic or hemorrhagic stroke occurring within 7–365 days of the index event. Recurrence was confirmed by clinical deterioration accompanied by radiological evidence of a new lesion that is distinct from the initial infarct and not attributable to hemorrhagic transformation, drug toxicity, or concurrent acute brain injury (Alraddadi et al., 2025). Secondary outcomes included stroke severity, functional capacity on admission and at discharge, mortality rate, hemorrhagic transformation, and stroke-related complications within 1 year of the index stroke. Stroke severity was assessed using NIHSS, while functional capacity was evaluated using the modified Rankin Scale (mRS) (Alamri et al., 2024).

### 2.4 Data collection

Collected data included demographic information and clinical characteristics, including the Trial of ORG 10172 in Acute Stroke Treatment (TOAST) classification, tissue plasminogen activator (tPA) therapy, prior history of stroke, mechanical thrombectomy, intensive care unit (ICU) admission, comorbidities such as HTN, DM, dyslipidemia, atrial fibrillation (AF), dementia, and depression. Stroke-related complications were also documented, including motor impairments, aphasia, dysarthria, pneumonia, cerebral edema, deep vein thrombosis-pulmonary embolism (DVT-PE), and impaired consciousness (defined as a Glasgow Coma Scale; GCS 3–14).

### 2.5 Propensity score matching

This study utilized variables such as age, gender, HTN, DM, dyslipidemia, AF before stroke, history of stroke, and stroke subtype for matching purposes. These variables were employed in the PSM process to obtain two well-balanced patient groups: one comprising patients exposed to steroids and the other serving as a control group. Propensity scores were determined using a logistic regression model, and a 1:2 nearest-neighbor matching algorithm without replacement was applied to match the patients (Austin, 2011).

### 2.6 Statistical analysis

Descriptive statistics were utilized to summarize the baseline characteristics of the study sample before and after PSM. Continuous variables were presented as the mean ± standard deviation (SD) or median (interquartile range), while categorical variables were presented as frequencies and percentages. Group comparisons were performed using paired t-tests for continuous variables and chi-squared tests for categorical variables.

Univariate and multivariate conditional logistic regression analyses were conducted to assess the association between steroid exposure and stroke recurrence after adjusting for potential confounders. Odds ratios (ORs) with 95% confidence intervals (CIs) were reported. Conditional models were adjusted for aphasia, dysarthria, pneumonia, DVT–PE, impaired consciousness, tPA, mechanical thrombectomy, and ICU admission. Although the TOAST classification was included in the PSM and remained significantly different between the exposure groups, it was excluded from the final regression models due to sparse data, which led to model non-convergence. To assess the impact of missing covariate data (395 patients excluded) and small sample size, we performed five‐fold multiple imputation by chained equations on the whole 1,320‐patient cohort. Each imputed dataset underwent the same 1:2 greedy‐caliper PSM and conditional logistic regression as the complete‐case analysis. Estimates were then pooled across imputations using Rubin’s rules. All statistical analyses were performed using SAS statistical software (version 9.4; SAS Institute, Inc., Cary, NC, United States). A *p*-value of <0.05 was considered statistically significant.

## 3 Results

### 3.1 Baseline characteristics

The initial study sample consisted of 925 patients, of whom 85 (9.19%) had been exposed to steroids. Following PSM, the final analysis included 254 patients, with 85 assigned to the steroid exposure group. Baseline characteristics of the study sample before and after PSM are summarized in Table 1. Post-PSM comparisons revealed no significant differences between the steroid and non-steroid groups in terms of mean age, gender distribution, or prevalence of comorbidities, including HTN, DM, dyslipidemia, AF, dementia, and depression (*p* > 0.05). However, significant differences were observed in the TOAST classification, particularly in subtypes 1 (Large Artery Atherosclerosis) and 5 (Undetermined Etiology) (*p* < 0.0001). Additionally, no significant differences were found between the groups regarding history of stroke (*p* = 0.7559).

**TABLE 1:** Basic characteristics of study sample before and after propensity score matching

Characteristics	Before PSM	After PSM
Steroid (Yes), n (%)	85 (9.19%)	85 (33.46%)
Age, mean ± SD^a^	64.75 ± 13.02	64.75 ± 13.02
Male, n (%)^a^	579 (62.59%)	116 (45.67%)
TOAST, n (%)^a^		
1 (Large artery atherosclerosis)	430 (51.68%)	120 (47.24%)
2 (Small vessel occlusion)	197 (23.68%)	63 (24.80%)
3 (Cardioembolism)	57 (6.85%)	24 (9.45%)
4 (Other determined etiology)	34 (4.09%)	20 (7.87%)
5 (Undetermined etiology)	114 (13.70%)	27 (10.63%)
History of stroke, n (%)^a^	328 (35.46%)	90 (35.43%)
tPA therapy, n (%)	96 (10.39%)	15 (5.91%)
Mechanical thrombectomy, n (%)	47 (5.09%)	10 (3.94%)
ICU admission, n (%)	287 (31.13%)	95 (37.55%)
Comorbidities, n (%)^a^		
HTN	740 (80.00%)	207 (81.50%)
DM	672 (72.65%)	195 (76.77%)
Dyslipidemia	426 (46.05%)	123 (48.43%)
Atrial fibrillation	90 (9.73%)	47 (18.50%)
Dementia	44 (4.76%)	17 (6.69%)
Depression	131 (14.19%)	51 (20.08%)
Stroke-related complications, n (%)		
UL Motor Impairment	419 (46.92%)	112 (48.91%)
UR Motor Impairment	381 (42.67%)	115 (50.00%)
LL Motor Impairment	411 (46.13%)	111 (48.68%)
LR Motor Impairment	365 (41.06%)	110 (48.25%)
Aphasia	260 (28.17%)	69 (27.27%)
Dysarthria	544 (58.81%)	141 (55.51%)
Pneumonia	210 (22.70%)	83 (32.68%)
Cerebral edema	81 (8.78%)	34 (13.39%)
DVT-PE	87 (9.41%)	40 (15.75%)
Impaired consciousness	249 (26.92%)	78 (30.71%)
Outcome, n (%)		
Hemorrhagic transformation	110 (11.92%)	47 (18.58%)
Stroke recurrence	160 (17.33%)	63 (24.80%)
Death	191 (20.69%)	75 (29.53%)

PSM, propensity score matching; SD, standard deviation; TOAST, Trial of ORG, 10172 in Acute Stroke Treatment; tPA, tissue Plasminogen Activator; ICU, intensive care unit; HTN, hypertension; DM, diabetes mellitus; UL, upper left; UR, upper right; LL, lower left; LR, lower right; DVT-PE, deep vein thrombosis-pulmonary embolism.

^a^ Variables were included in the propensity score matching.

### 3.2 Steroid exposure and stroke severity

Stroke severity on admission and at discharge was assessed using the NIHSS and mRS scores. Patients in the steroid exposure group had significantly lower NIHSS scores at both admission (median [25th–75th percentiles]: 5 [1–8] vs. 6 [3–10], *p* = 0.0087) and discharge (median 1 [IQR: 0–4.5] vs 4 [IQR: 2–9], *p* = 0.0001). In contrast, mRS scores at discharge were significantly higher in the steroid exposure group (5 [4–5] vs. 4 [3–5], *p* = 0.0004), Table 2.

**TABLE 2:** Comparisons of stroke severity at admission and discharge between patients with and without steroid exposure.

	Steroid (yes)	Steroid (no)	*P*
NIHSS on admission	5 (1–8)	6 (3–10)	**0.0087**
NIHSS at discharge	1 (0–4.5)	4 (2–9)	**0.0001**
mRS on admission	4 (2–5)	4 (1–5)	0.5508
mRS at discharge	5 (4–5)	4 (3–5)	**0.0004**

NIHSS, national institutes of health stroke scale; mRS, modified Rankin Scale.

Data are shown as median (25th–75th percentiles).

### 3.3 Stroke symptoms and complications

The results of the univariate analysis examining the association between steroid exposure and stroke symptoms or complications are presented in Table 3. Steroid exposure was significantly associated with a reduced likelihood of aphasia (OR, 0.33; 95% CI: 0.17–0.67, *p* = 0.0020) and dysarthria (OR, 0.51; 95% CI: 0.30–0.88, *p* = 0.0149). Conversely, steroid exposure was linked to an increased risk of several complications, including pneumonia (OR: 2.08, 95% CI: 1.22–3.55, *p* = 0.0071), DVT-PE (OR: 2.70, 95% CI: 1.30–5.62, *p* = 0.0079), and impaired consciousness (OR: 1.80, 95% CI: 1.06–3.04, *p* = 0.0303).

**TABLE 3:** Unadjusted analysis of stroke symptoms and complications based on steroid exposure.

Symptoms	OR	95% CI	*P* ^a^
UL Motor Impairment	1.46	0.78–2.74	0.2428
UR Motor Impairment	0.57	0.31–1.05	0.0723
LL Motor Impairment	1.13	0.62–2.06	0.6832
LR Motor Impairment	0.75	0.42–1.34	0.3245
Aphasia	0.33	0.17–0.67	**0.0020**
Dysarthria	0.51	0.30–0.88	**0.0149**
Complications			
Pneumonia	2.08	1.22–3.55	**0.0071**
Cerebral edema	1.12	0.51–2.42	0.7893
DVT-PE	2.70	1.30–5.62	**0.0079**
Impaired consciousness	1.80	1.06–3.04	**0.0303**

OR: odds ratio; CI, confidence interval; UL, upper left; UR, upper right; LL, lower left; LR, lower right; DVT-PE, deep vein thrombosis-pulmonary embolism.

^a^ *P* was estimated by univariate conditional logistic regression.

### 3.4 Steroid exposure and stroke outcome

Conditional logistic regression analysis was conducted to assess the relationship between steroid exposure and stroke outcomes while accounting for potential confounders, Table 4. In the unadjusted model, steroid exposure was associated with an increased risk of stroke recurrence (OR: 1.98, 95% CI: 1.01–3.87, *p* = 0.0471). However, after adjusting for confounders, this association lost significance (OR: 1.14, 95% CI: 0.44–2.96, *p* = 0.7874). Additionally, steroid exposure was not significantly associated with hemorrhagic transformation or mortality in either the unadjusted or adjusted models. Furthermore, the pooled ORs after multiple imputation (Supplementary Appendix Table 1) closely mirrored the complete‐case analysis. In the multiple‐imputation sensitivity analysis, the adjusted ORs were (aOR: 1.25, 95% CI 0.32–4.90; *p* = 0.7227) for hemorrhagic transformation, (aOR: 1.61, 95% CI 1.00–2.58; *p* = 0.0499) for stroke recurrence, and (aOR: 1.20, 95% CI 0.69–2.10; *p* = 0.5040) for death.

**TABLE 4:** Association between steroid exposure and stroke outcome.

	Unadjusted model			Adjusted model^a^		
Outcome	OR	95% CI	*P*	OR	95% CI	*P*
Hemorrhagic transformation	0.70	0.34–1.44	0.3262	0.87	0.28–2.73	0.8070
Stroke recurrence	1.98	1.01–3.87	**0.0471**	1.14	0.44–2.96	0.7874
Death	1.36	0.78–2.38	0.2748	0.75	0.34–1.67	0.4842

OR, odds ratio; CI, confidence interval.

^a^ Conditional logistic regression models were adjusted for aphasia, dysarthria, pneumonia, DVT-PE, impaired consciousness, tissue plasminogen activator, mechanical thrombectomy, and ICU, admission.

## 4 Discussion

This retrospective hospital-based observational study investigated the association between prior steroid use and stroke recurrence and outcomes in the Saudi Arabian population. Our findings revealed that patients exposed to steroids presented with lower NIHSS scores on admission, indicating the potential protective effect of steroids on initial stroke severity. However, despite the initial benefit, the steroid group exhibited significantly higher mRS scores at discharge, reflecting poorer functional outcomes, Table 2. These results suggest a paradoxical relationship between steroid exposure and stroke recovery. Glucocorticoids, a class of steroid hormones, are extensively utilized for their anti-inflammatory properties and capacity to modulate immune responses. It can reduce acute inflammation and potentially mitigate neurological damage (Duque Ede and Munhoz, 2016). For instance, glucocorticoids have been shown to inhibit the expression of pro-inflammatory cytokines and reduce blood-brain barrier permeability, thereby protecting neural tissue from further injury (Duque Ede and Munhoz, 2016), which could explain the lower NIHSS scores observed on admission in the steroid group. However, the effects of glucocorticoids on the central nervous system are complex and can vary depending on factors such as dosage, duration of exposure, and the specific neurological context. While acute administration may confer neuroprotective benefits, chronic exposure to glucocorticoids has been associated with pro-inflammatory effects and potential neurotoxicity (Duque Ede and Munhoz, 2016). Studies have indicated that prolonged glucocorticoid exposure can lead to neuronal damage in regions like the hippocampus, potentially impairing cognitive functions (Sorrells and Sapolsky, 2007). These adverse effects could contribute to the observed higher mRS scores at discharge, reflecting poorer functional outcomes.

The analysis of steroid exposure in stroke patients revealed a complex relationship between steroid use, stroke symptoms, complications, and outcomes. The univariate analysis indicated that steroid exposure was significantly associated with a reduced likelihood of aphasia and dysarthria. This suggests that steroids may confer neuroprotective effects, potentially by attenuating cerebral inflammation and reducing neuronal damage in language-related brain regions. However, these benefits were counterbalanced by an increased risk of complications such as pneumonia, DVT-PE, and impaired consciousness. These risks align with existing research showing that glucocorticoids can significantly increase infection susceptibility. A previously published study found that higher doses of glucocorticoids are associated with a substantially greater risk of serious infections than other immunomodulatory therapies. Notably, even low-dose treatment carries a high risk of infection (Riley and George, 2021). A large population-based cohort study further supports these findings, reporting a two-to-six-fold increase in bacterial, viral, and fungal infections among individuals receiving oral glucocorticoids compared to non-users (Fardet et al., 2016). Additionally, the heightened incidence of DVT-PE may be attributed to glucocorticoid-induced hypercoagulability. Several studies have indicated an elevated risk of venous thromboembolism in patients exposed to glucocorticoids, which could explain the observed association (Sundbøll et al., 2018).

In the unadjusted model, steroid exposure was associated with an increased risk of stroke recurrence, Table 4. However, after adjusting for potential confounders, this association lost significance. This suggests that the initial observed risk may be attributed to underlying factors rather than steroid use itself. One possible explanation is that steroid therapy is more commonly prescribed in patients with chronic inflammatory or autoimmune diseases, which are independently associated with higher vascular risk. Therfore, the unadjusted association likely reflected this elevated baseline risk rather than a direct causal role of steroids. After controlling for these confounders, the observed effect size diminished. This supports the notion that steroid use may serve as a proxy for disease severity rather than a primary risk factor for recurrence. Supporting this, a nationwide cohort study found no significant association between current glucocorticoid use and recurrent ischemic stroke (Sundbøll et al., 2018). Similarly, the current study found no significant association between steroid exposure and hemorrhagic transformation or mortality in both unadjusted and adjusted models. This aligns with existing literature, which indicates that glucocorticoid use does not significantly impact the risk of hemorrhagic stroke (Sundbøll et al., 2018) or mortality in acute ischemic stroke patients (Poungvarin, 2004).

This study had several limitations that should be acknowledged when interpreting the findings. Firstly, the retrospective design and reliance on medical records may introduce information bias due to incomplete or inaccurate data. Additionally, the study’s limited sample size and inclusion of only patients from Saudi Arabia may have introduced selection bias and reduced generalizability. In addition, unmeasured confounders, such as the underlying indications for steroid therapy (e.g., autoimmune or respiratory diseases) and undocumented comorbidities, may have influenced the observed associations with stroke recurrence and outcomes, potentially leading to overestimation or underestimation of the true effect of steroid exposure. Furthermore, AF was included in the PSM; however, given its established association with stroke recurrence and the limited sample size, residual confounding may still be present. Moreover, the significant differences observed in the TOAST classification, particularly among patients with stroke in subtype 1 (Large Artery Atherosclerosis), could have impacted the results, considering the higher likelihood of stroke recurrence due to Large Artery Atherosclerosis-type ischemic stroke (Lovett et al., 2004). Additionally, the absence of detailed information on the duration and dosage of steroid therapy precluded a more nuanced analysis of the dose-response relationship and its impact on stroke outcomes and stroke recurrence.

Future research should prioritize prospective studies with larger, more diverse cohorts and comprehensive data on steroid use, including precise dosing regimens, treatment durations, and indications. These studies must use robust databases that systematically record these parameters to clarify their role in stroke recurrence and outcomes, guiding individualized treatment strategies. Such studies would also help validate our findings and elucidate the mechanisms linking steroid exposure to stroke outcomes. Moreover, investigating the differential effects of various types of steroids and alternative anti-inflammatory therapies on stroke recurrence could provide critical insights into optimizing treatment strategies. These efforts could lead to more personalized and effective approaches to the management of stroke patients.

## 5 Conclusion

In conclusion, the present study showed that while steroids may reduce initial stroke severity, steroids can potentially increase the mRS score and worsen the overall functional capacity. Moreover, steroid patients are at higher risk of developing more complications compared to the control group. Further prospective studies with larger sample sizes and detailed data on steroid use and stroke outcomes are required to fully assess the effects of steroids.

## Data Availability

The original contributions presented in the study are included in the article/Supplementary Material, further inquiries can be directed to the corresponding author.
