# Supplementary material for: Effects of previous steroid treatment on ischemic stroke outcomes: a propensity score-matched hospital analysis
Source: Front Pharmacol. 2025 Jun 17;16:1615170. doi: 10.3389/fphar.2025.1615170 (PMC12209245; doi:10.3389/fphar.2025.1615170)
Supplement: Supplementary file 1 [file Table1.docx]

**Appendix Table 1. Pooled Unadjusted and Adjusted Odds Ratios (95% CI) for Hemorrhagic Transformation, Stroke Recurrence, and Death Associated with Steroid Exposure**

|  | **Unadjusted Model** | | | **Adjusted Model ^a^** | | |
| --- | --- | --- | --- | --- | --- | --- |
| **Outcome** | **OR** | **95% CI** | ***P*** | **OR** | **95% CI** | ***P*** |
| **Hemorrhagic transformation** | 1.19 | 0.63-2-22 | 0.5689 | 1.25 | 0.32-4.90 | 0.7227 |
| **Stroke recurrence** | 1.68 | 1.20-2.35 | 0.0024 | 1.61 | 1.00-2.58 | 0.0499 |
| **Death** | 1.39 | 1.01-1.91 | 0.0406 | 1.20 | 0.69-2.10 | 0.5040 |

OR: odds ratio; CI: confidence interval
 Conditional logistic regression models were run in each of five multiple imputation datasets and pooled using Rubin’s rules; ^a^ all models were adjusted for aphasia, dysarthria, pneumonia, DVT-PE, impaired consciousness, tPA, mechanical thrombectomy, and ICU admission.
